# Review of Technologies and Materials Used in High-Voltage Film Capacitors

**DOI:** 10.3390/polym13050766

**Published:** 2021-02-28

**Authors:** Olatoundji Georges Gnonhoue, Amanda Velazquez-Salazar, Éric David, Ioana Preda

**Affiliations:** 1 Department of Mechanical Engineering, École de technologie supérieure, Montreal, QC H3C 1K3, Canada; amanda.velazquez-salazar.1@ens.etsmtl.ca (A.V.-S.); eric.david@etsmtl.ca (É.D.); 2 Energy Institute—HEIA Fribourg, University of Applied Sciences of Western Switzerland, 3960 Sierre, Switzerland; ioana.preda@hefr.ch

**Keywords:** high-voltage capacitors, resin, dielectric film

## Abstract

High-voltage capacitors are key components for circuit breakers and monitoring and protection devices, and are important elements used to improve the efficiency and reliability of the grid. Different technologies are used in high-voltage capacitor manufacturing process, and at all stages of this process polymeric films must be used, along with an encapsulating material, which can be either liquid, solid or gaseous. These materials play major roles in the lifespan and reliability of components. In this paper, we present a review of the different technologies used to manufacture high-voltage capacitors, as well as the different materials used in fabricating high-voltage film capacitors, with a view to establishing a bibliographic database that will allow a comparison of the different technologies

## 1. Introduction

High-voltage films capacitors are important components for networks and various electrical devices. They are used to transport and distribute high-voltage electrical energy either for voltage distribution, coupling or capacitive voltage dividers; in electrical substations, circuit breakers, monitoring and protection devices; as well as to improve grid efficiency and reliability. Impregnated either with gas or oil, they can be categorized into six different classes, namely high-power capacitors, high-voltage capacitors, energy storage capacitors, starting capacitors, filter capacitors and discharge capacitors [1]. Table 1 shows the history of the development of capacitors.

One particular category, namely capacitors used in high-voltage applications, is of particular interest in the present study, given the key role these devices play in improving grid efficiency by stabilizing voltage levels. The dielectric materials used for these capacitors play a key role in their performance and long-term reliability. Prior to the 1970s, impregnated kraft paper was the main capacitor dielectric, usually used in combination with mineral oil or polychlorinated biphenyl (PCB) as an impregnating liquid, however today these components are often manufactured using polymeric films. Thanks to their low dissipation factor, high dielectric strength, good stability and high availability, polymer films have gradually replaced the kraft paper used in capacitors. The switch from paper to polymer film has also shortened the capacitor production process by reducing the drying time required before impregnation [2,3]. Furthermore, as with polymer technology, capacitor manufacturing technologies have evolved over time. Film capacitors have, thus, been fabricated using polyethylene (PE), polystyrene (PS), polytetrafluoroethylene (PTFE), polyethylene terephthalate (PET) and polycarbonate (PC) films, and most recently, biaxially-oriented polypropylene (BOPP), which is the current choice for capacitors used in high-voltage applications [4,5]. Additionally, mixed capacitors associating paper layers with polymeric layers have been used with different impregnating components, as shown in Table 2.

**Table 1:** History of capacitor development [6].

Capacitors	Years
Water in Leyden Jar	1746
Franklin’s glass–metal foil	1750
Paper	1876
Electrolytic capacitor	1887
Wax paper–metal foil	1876
Self-clearing capacitor	1900
Mica	1909
Wound electrolytic capacitor	1927
Lacquer on paper	WWII
Polymeric films	Starting from 1954

Table 2 shows the evolution over time of the different power capacitors, as well as their electrical and dielectric characteristics.

## 2. Design of High-Voltage Film Capacitors

Film capacitors are manufactured in the form of a winding using a capacitor winding machine, or in the form of a stack of dielectric films. These two manufacturing technologies are also respectively known as coil technology and the stacking technique [7,8]. Four different types of film capacitors can be found in both technologies, namely mixed capacitors, all-paper capacitors, all-film capacitors and metallized film capacitors [9]. Furthermore, depending on the choice of electrodes used, capacitors can take on two forms: dielectric armature capacitors and metallized capacitors. Usually, armature capacitors are used for very high-current applications, while metallized film capacitors are used for low-current applications.

Among these different film capacitors, supercapacitors should also be mentioned. Unlike their high-voltage counterparts, they can use conducting polymers as electrodes, disconnected by a separator and placed in an electrolyte, a chemical liquid containing a mixture of positive and negative ions dissolved in water. Their particular characteristics will be further discussed in Section 2.3.

Mixed capacitors consist of a sheet of paper used in conjunction with polymeric films and impregnated with a non-chlorinated liquid dielectric, while all-film capacitors only use polymeric films, also impregnated with a non-chlorinated liquid dielectric.

All metallized film capacitors consist of a layer of metallized film and can be impregnated (or not) with a liquid or gaseous dielectric [10]. The commonly used technologies will be further detailed in this section.

### 2.1. Metallized Electrode Capacitors

**Figure 1:**
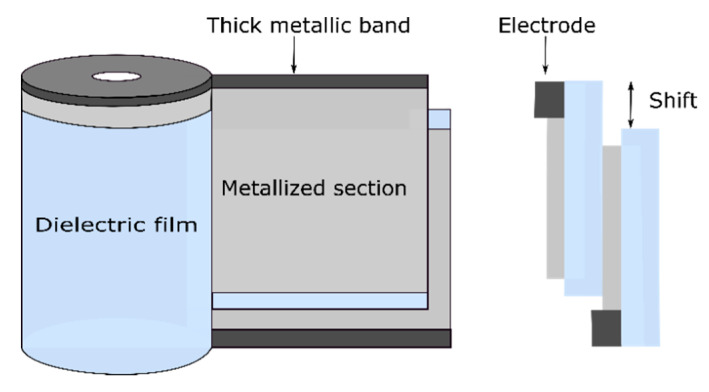
Single-section metallized capacitor.

The first step in building a metallized film capacitor is physical vapor deposition under vacuum of a very thin layer (10 to 100 nm) of metal, such as aluminum, zinc or zinc–aluminum, on one side (evaporated to the surface) of a roll of polymer film [7]. Sometimes, small amounts of other alloy metals are added to prevent corrosion. There are five main types of metallized capacitor designs, namely the dry metallized film design (Figure 1), liquid-impregnated–gas-impregnated metallized film design, cellulosic paper design, liquid-impregnated metallized polymer film design (mixed or all-film dielectric) and stacked metallized film design (Figure 3). The coiling of the metallized capacitors is achieved by winding two sheets of metallized film on a hard, insulating cylindrical core (Figure 5). During the manufacturing process, the rolls are wound as tightly as possible to prevent the formation of air vacuoles or cavities [11].

Table 3 summarizes the main bibliographic references, focusing on metallized capacitor technologies.

The advantage of wire -wound metallized capacitors with a metallic film is that they take up two to three times less space than those carrying armatures, and additionally they exhibit self-healing behavior. The self-healing effect can be explained as follows: in the event of a defect in the dielectric material, the energy given off by the discharge is generally sufficient to vaporize the metallization around the defect, and therefore to electrically isolate it. The capacitor loses part of its capacitance but regains its isolation and continues to operate. Connections are made by metallization using the shoopage technique (Figure 3), which consists of depositing a molten metal on both faces of the capacitors [7,8]. The shoopage is carried out by spraying metal, usually zinc or a zinc alloy, in order to connect to the metallization of the film [4].

**Figure 2:**
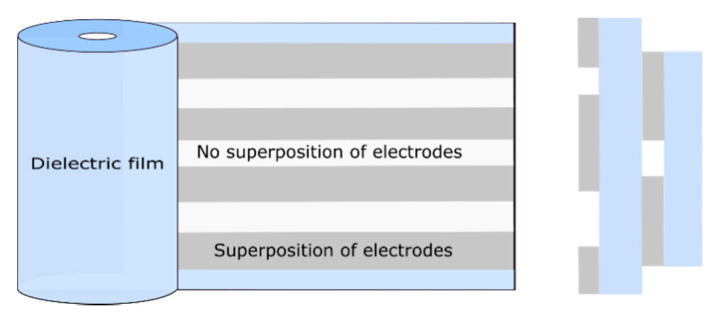
Metallized multisection capacitor [16].

According to [2,10], it is recommended that before metallization, the film should undergo a corona surface treatment in order to facilitate its adhesion to the metallization (however, this action is not mandatory). After this treatment, the film is metallized in a high-speed vacuum metallizer. This is usually followed by the application of a voltage higher than the nominal one in order to ensure that all defects created during manufacture are removed from the winding. This may lead to a slight decrease in capacitor capacity, but after the operation, the capacitor will remain within specifications. A known limitation in the fabrication process arises from the choice of connections, as they constitute the weak point of metallized film capacitors. Furthermore, the two films must each have a margin to avoid a short circuit between the electrodes [7]. A distinction is made between metallized capacitors wound with a single section and those with multiple sections (Figure 1 and Figure 2). Figure 1 and Figure 2 respectively present the geometry of a metallized single-section capacitor and of a metallized multisection capacitor.

Coils of multisection metallized film can be made in the same capacitors. In this case, the sections are transverse or longitudinal.

The main advantage of multisection metallized capacitors comes from lowering the inductance and resistance values of the winding [16]. Figure 2 shows the configuration of the longitudinal multisection metallized capacitor and the arrangement of the metallization on the dielectric film. Furthermore, to manufacture the connections of multisection metallized capacitors, tabs are inserted in the center of the capacitors and on the outer edge in order to contact the first and last sections of the winding [2].

The performance of a metallized film capacitor can also be improved by using mixed dielectric layers, i.e., rough paper and film. In this case, two layers of paper are metallized on one side and a film is stacked between the two paper layers. For this assembly, there is no need to metallize the film. The paper could have a rough surface, in which case it would have the advantage of easily allowing the liquid impregnator to penetrate and fill the space between the film–paper and paper–electrode sections. The film may also have a rough surface, however using a film polymer with a rough surface when manufacturing a film capacitor is not mandatory [11].

**Figure 3:**
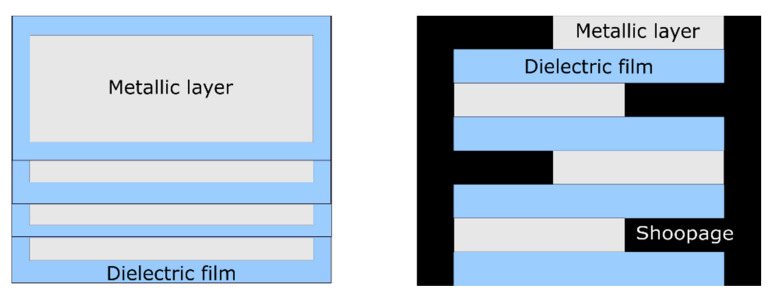
Stacked metallized capacitors.

**Figure 4:**
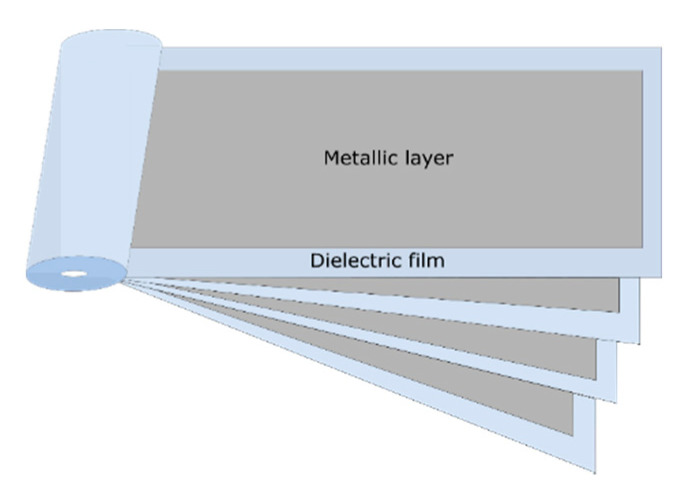
Metallized capacitor coil.

Stacked-film metallized capacitors consist of a stack of elementary capacitors connected in parallel [8]. This particular assembly is suitable for applications requiring good compaction. The manufacturing technique that uses stacked film capacitors (Figure 3) is the same as that using wound capacitors (Figure 4), however in the case of metallized capacitors, two insulating films with metallization (aluminum layer 20 to 30 nm thick) are used, whereas with wound capacitors, and as shown in Figure 4, the film–metallized electrode assembly is wound on a wheel (winder) measuring about 80 cm in diameter [7]. Figure 3 and Figure 4 respectively show the configuration of stacked metallized and metallized capacitor coils. They present the metallized layers, the dielectric film and the location where the electrical connections are created (shoopage).

### 2.2. Foil Capacitors

#### Foil–Film Capacitor Fabrication

**Figure 5:**
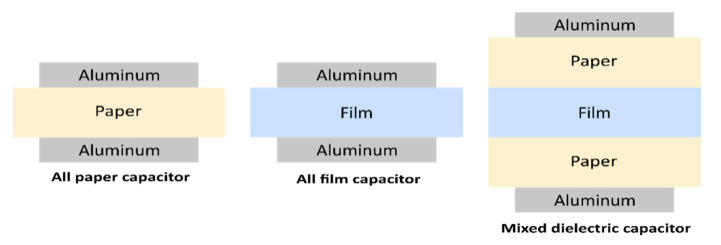
Different types of foil–film capacitors.

As shown in Figure 5, foil capacitors consist of two thin metal films (aluminum or zinc), which are only 5 to 6 µm thick, and are separated by an insulating medium and impregnated (or not) with a liquid dielectric. Winding electrodes and films form the coil. The aluminum electrode must be perfectly clean. Specifically, an adequate treatment must be used to eliminate all lubricating products used during rolling. In addition, it must have good regularity, a satisfactory mechanical strength and must not have any folds or tears [10]. The connections of wound foil capacitors can be made either by inserting several metal tabs in contact with each armature or by making each armature protrude through the insulating material, after which the connections are welded [10].

When capacitors are manufactured using the foil–film technique, they are subjected to a drying treatment to remove the moisture present. This is then followed by encapsulation of the capacitor. Encapsulation can be separated into several stages: preparation of the resin or oil, degassing, transfer and finally crosslinking when a resin or a polymer is used.

When preparing the encapsulation media, care should be taken to avoid introducing excessive amounts of air, which will have to be extracted in the following steps. One solution involves using an automatic mixing machine, which not only allows the resin and the different products to be mixed in appropriate proportions, but also does not introduce air into the mix. The containers used for the preparation of the resin must, therefore, remain closed when not in use in order to prevent any moisture penetration. Before being used, the resin is degassed under vacuum to remove air bubbles from the dielectric liquid, given that residual air bubbles are prone to exhibiting partial discharges that can cause a reduction in voltage withstand capability and premature aging of the capacitor [18].

Film–foil capacitors are available in several configurations, namely, wound foil–film, stacked foil–film, hybrid film–foil metallized combination, inserted tab and multisection film–foil set-ups. Table 4 shows different technologies and the main review articles focusing on each foil–film capacitor technology.

Stacked foil–film capacitors are configured similarly to stacked metallized capacitors, however metal electrodes are used in the latter, and as with multisection foil–film capacitors, they are not self-healing. Figure 5 shows different types of wound and stacked foil–film capacitors.

In the technology involving the use of combined metallized foil–film hybrid capacitors, one or more foil electrodes are used, along with a metallized film surface. Using these two configuration types in combination provides the joint advantages inherent to using both metallized capacitors and foil electrodes.

**Figure 6:**
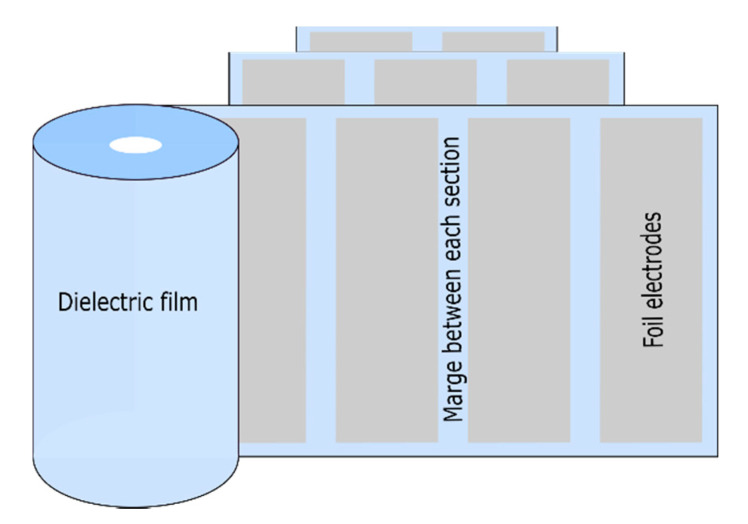
A multisection foil–film capacitor [19].

As shown in Figure 6, multisection foil–film capacitors consist of metal foil electrodes separated by one or more layers of dielectric material. They are wound in a spiral, and in this type of this capacitor, tabs are connected to the electrodes to establish connections. The main advantage of multisection foil–film windings is the resulting reduction in volume in the margin regions between the sections. Multisection foil–film capacitors allow several sections in series. This configuration is considered as a circuit for different capacitors in series. The main disadvantages of such capacitors are that they are not self-healing as metallized capacitors are and they have high equivalent series inductance (ESL) [19].

It is possible to achieve a configuration of multilayer capacitors using only polymers or nanocomposites (by introducing inorganic nanofillers into the polymer matrix). This technique improves the permittivity of composite materials. Different capacitor configurations can be obtained using composites, such as composite inorganic particles (polymers structured in sandwich configurations), composite inorganic particles (polymers structured in inverted sandwich configurations) and composite inorganic particles (polymers structured in gradient layers).The multilayer configuration using only polymers includes a configuration for all films with a sandwich structure and a configuration for all films in nanolayers. The sandwich structure composites are constructed by inserting an insulating layer with high dielectric strength between two layers with high dielectric constants. This configuration allows the electrical breakdown properties of the capacitors to be adjusted. Gradient-layer-structured inorganic particle–polymer composites improve the local electric field around the interfaces between the different phases. It also makes it possible to reduce the electric shafts between the interfaces of the different layers [20].

All-film sandwich capacitors can be configured by mixing two or more types of polymers to form fully polymeric materials. For example, nanolayered all-polymer films technology consists of manufacturing nanolayer polymer films using electrospinning in combination with hot pressing. Reverse-sandwich-structured inorganic particle–polymer composites are made by placing the insulating layers (such as the outer layers) near the electrodes. This design contributes to improving the charging–discharging efficiency of the capacitors [20].

Figure 6 shows the configuration of a foil–film multisection capacitor.

### 2.3. Supercapacitors

Supercapacitors are electrochemical capacitors with higher capacitance values than ordinary capacitors. Supercapacitors can be broken down into three types, depending on their electrode characteristics: double-layer capacitors (EDLC), which use carbon materials (such as activated carbon, carbon nanofibers, carbon airgels); pseudocapacitors, which use metal oxides or conductive polymers as electrodes; and hybrid capacitors, which have asymmetric electrodes (one electrostatic electrode and another electrochemical electrode).

The main difference between this type of capacitor and conventional, electrolytic capacitors or high-voltage capacitors is the actual capacitance value, since supercapacitors have a much higher capacitance (of the order of hundreds or thousands of Farads). Furthermore, the voltage level is low in supercapacitors compared to their high-voltage counterparts: while conventional capacitors can withstand high voltage levels, the voltage limit for supercapacitors is between 2.5 and 2.7 V. Last but not least, supercapacitors are used for storage with high power density and high-voltage capacitors are used for voltage distribution (as voltage dividers, for example). This characteristic is in line with their specific capacitance: supercapacitors have a very high capacitance, which ranges from a few Farads up to thousands of Farads, while high-voltage capacitors have extremely low capacitances (pF or nF).

Supercapacitors mainly consist of two electrodes, an electrolyte and a separator. It should be noted that electrodes in supercapacitors store more energy than is the case in a regular capacitor and that the electrolyte is a mixture of ions (positive and negative) dissolved in a liquid. The separator can exist in coiled form, and in the case of an organic electrolyte, can consist of polymer type. The separator serves to prevent short circuits between electrodes, as well as to allow ionic transfer between them. The separator must have high electrical resistivity and be very thin and chemically static to maintain the stability and conductivity of the electrolyte. For organic electrolytes, polymers or paper separators are used. A supercapacitor can operate in a temperature range of 70 °C to −20 °C. Compared to ordinary capacitors, supercapacitors are maintenance-free, environmentally friendly, totally pollution-free and carry a minimal risk of explosion [21,22,23,24,25].

Polymers such as poly(propylene oxide), poly(ethylene imine), thio-alkane, polystyrene, poly(vinyl alcohol), poly(vinylidene fluoride) (PVdf), poly(vinylidene carbonate), poly(acrylonitrile), poly(vinyl chloride), poly(vinylsulfone), poly(p-phenylene tereththalamide) and poly(vinylpyrolidone) are often used in supercapacitors. In the manufacture of electrolytes, hydrogels made with biopolymers such as crosslinked gelatin are highly hydrophilic and have better water holding capacity than those made with synthetic polymers. Hydrogels based on biopolymers such as genipin can also be used. Other hydrogel electrolytes such as poly(ethylene oxide) and poly(acrylate) potassium are also used [25].

## 3. Dielectric Materials Used in High-Voltage Capacitors

### 3.1. Dielectric Materials

Ideal dielectric materials are characterized by an absence of free charges in their constituent volume. However, the chains constituting a polymer material can experience a local displacement movement under the action of an external electrical field. When an electrical field is applied to a dielectric material, the constituent dipoles orient themselves with a delay in time. The orientation of these dipoles is manifested under the phenomenon of polarization. This mechanism of deformation of the distribution of electrical charges is called the phenomenon of polarization. Polarization is a physical quantity used in the study of the properties of dielectric materials. It designates the density of electrostatic dipoles. Its mechanism is characterized by an increase in the capacitor’s ability to store charges after the introduction of a dielectric.

**Figure 7:**
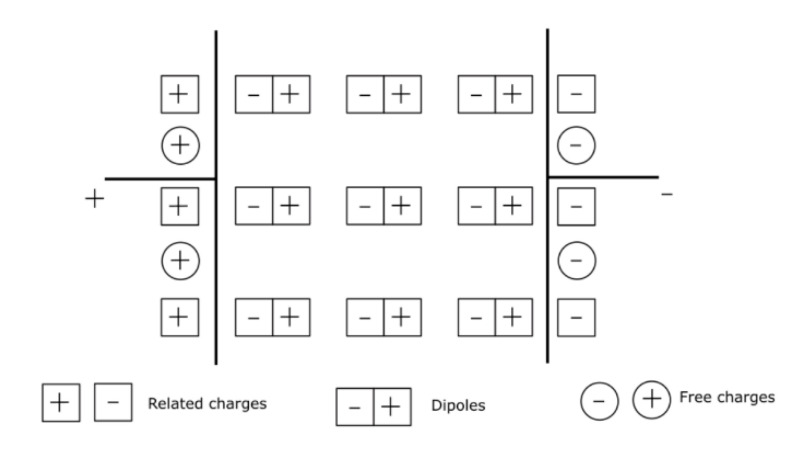
Illustration of polarization in a dielectric material.

There are four types of polarization that can be observed in polymeric materials: electronic, atomic, orientation and interfacial. As for the types of charges on the capacitor electrodes, they can be either free charges related to maintenance of the vacuum voltage or fixed charges, which are required to compensate for the phenomenon of polarization. Figure 7 presents this phenomenon in a dielectric material. It shows the free charges (negative and positive charges) on the terminals of the electrodes, as well as related charges (dipoles).

An electric dipole is formed by two charges *Q* of opposite signs separated by a distance *l*. The electric dipole moment is defined by:

(1) $$\vec{p}=q\vec{l}$$

The polarization due to these dipoles corresponds to the sum of vectors of all the dipoles per unit volume. The polarization $\vec{P}$ can be defined by:

(2) $$\vec{P}=\frac{1}{\Omega }\underset{i\in \Omega }{\sum }\vec{P}i$$

The units of polarization are C/m^2^. Consider a linear, homogeneous and isotropic dielectric material. If an electric field is applied to it, the relationship between $\vec{P}$ and $\vec{E}$ is linear and the polarization can be written by the following expression:

(3) $$\vec{P}=\varepsilon_{o}\chi \vec{E}$$

where *χ* is the electrical susceptibility of the material. If the electrical field is variable over time, a practical way to account for the delay between polarization and the electrical field (causality principle) is to use complex algebra. The electrical susceptibility is then a complex number and is dimensionless. It can be written as follows:

(4) $$\chi *\left(\omega \right)=\chi^{\prime }\left(\omega \right)-j\chi^{\prime \prime }\left(\omega \right)$$

If the polarization is due to a dipole relaxation mechanism having a time constant *τ*, a simple way (but which rarely corresponds to experimental observations) to express the variation of the complex susceptibility as a function of the frequency is to use Debye’s equations:

(5) $$\chi^{\prime }\left(\omega \right)=\chi_{\infty }+\frac{\left(\chi_{s}-\chi_{\infty }\right)}{1+\omega^{2}\tau^{2}}$$

(6) $$\chi^{\prime \prime }\left(\omega \right)=\frac{\left(\chi_{s}-\chi_{\infty }\right)\omega \tau }{1+\omega^{2}\tau^{2}}$$

The equivalent Debye’s equations for the complex permittivity as a function of frequency are:

(7) $$\varepsilon^{\prime }\left(\omega \right)=\varepsilon_{\infty }+\frac{\left(\varepsilon_{s}-\varepsilon_{\infty }\right)}{1+\omega^{2}\tau^{2}}$$

(8) $$\varepsilon^{\prime \prime }\left(\omega \right)=\frac{(\varepsilon_{s}-\varepsilon_{\infty })\omega \tau }{1+\omega^{2}\tau^{2}}$$

where $\varepsilon_{s}$ is the static electrical permittivity (for f = 0), $\varepsilon_{\infty }$ is the electrical permittivity at infinite frequency and ω is the pulsation *ω* = 2πf.

The real and imaginary permittivities are influenced by temperature as well as by frequency. The relaxation time depends on the temperature, with a dependence which often follows the form of an Arrhenius law:

(9) $$\tau =\tau_{0}e^{e_{a}/kT}$$

with $e_{a}$ being the activation energy and k bring the Boltzmann’s constant.

In polymers, the different molecular movements generated by the polarization mechanisms produce relaxation phenomena. These relaxations are classified in order of increasing frequency of occurrence at a fixed temperature or in order of decreasing temperature at a fixed frequency, and are identified by the successive letters of the Greek alphabet (*α*, *β*, *γ*). The *α* relaxation generally corresponds to the vibration and reorientation of the main chains of the crystalline regions in semicrystalline polymers and to the movements of the side chains during the glass transition for amorphous polymers. The *β* relaxation corresponds to the movement of the side chains at the glass transition for semicrystalline polymers and to localized movements of the side groups for amorphous polymers. The *γ* relaxation corresponds to the localized movements of the side groups of the amorphous regions for semicrystalline or amorphous polymers.

**Figure 8:**
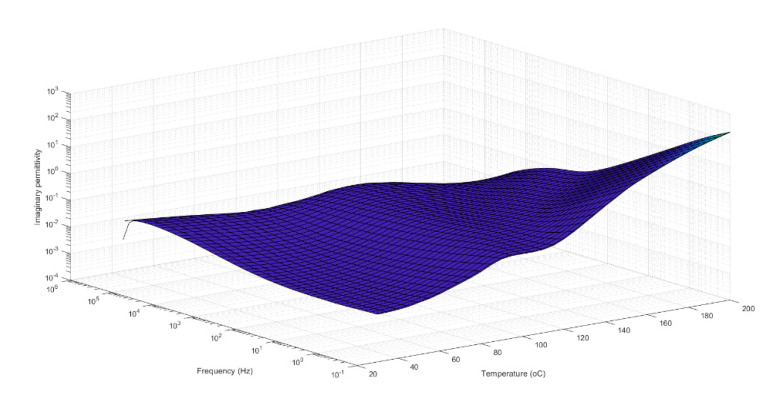
Dielectric losses of PET as functions of temperature and frequency.

**Figure 9:**
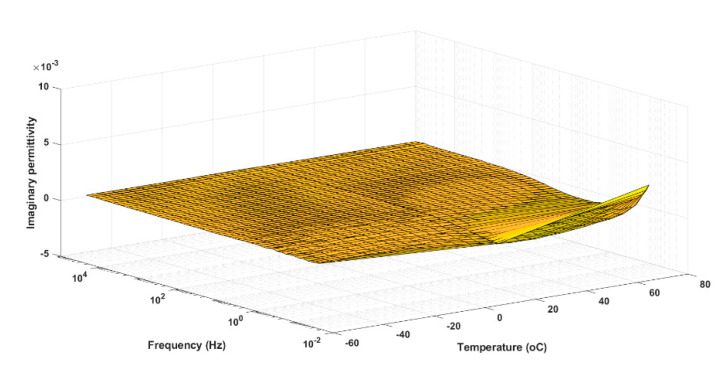
Dielectric losses of low-density polyethylene (LDPE) as functions of temperature and frequency.

Figure 8 and Figure 9 respectively show the responses of the dielectric losses of a typical polar polymer (PET) and of a non-polar polymer (PE). On the one hand, in Figure 8, local maxima associated with the α and β relaxation mechanisms can be observed. These peaks are due to the movements of the main and side chains or rotational movements of groups of the polymer. In Figure 8, the α relaxation mechanism is illustrated between frequencies from 1 to 10 (Hz) at temperatures ranging from 80 to 140 °C, and the presence of the β relaxation mechanism is manifested at high frequency. On the other hand, only the DC conductivity can be observed in the relaxation spectra of polyethylene at high temperature and low frequencies (the conduction phenomenon is manifested in Figure 8 and Figure 9 by an increase in dielectric losses when the frequency decreases).

Dielectric breakdown measurement is a destructive diagnostic method used to characterize the dielectric strength of materials. It is an irreversible destructive phenomenon in solid insulation materials. This type of measurement is typically based on the application of an increasing electrical field until the solid insulation breaks. The measurements can be made with an oil tester using degassed insulating oil to avoid flashover. To perform the measurements, several electrode geometries, such as spherical or mushroom-type electrodes, can be used. It is important to recognize that this test must observe certain standards (for example, the ASTM D149 or IEEE Std-930 standards), since the obtained results are highly dependent on the measurement procedure. The breakdown field is calculated by dividing the voltage at which the sample has broken down by the thickness of the same sample. The Weibull cumulative probability function is most often used to process experimental data. The expression of the two-parameter Weibull distribution is given by:

(10) $$P\left(E\right)=1-exp\left(-{\left(\frac{E}{\alpha }\right)}^{\beta }\right)$$

where *P*(*E*) represents the probability of failure and *E* is the experimental dielectric strength. The form factor, *β*, indicates the dispersion of the data, while the scale parameter, *α*, indicates the electrical field, for which the probability of breakdown is 63.2%. This last parameter is commonly used as the material characteristic breakdown strength. The estimators of *α* and *β* for a given sample can be calculated using a weighted least squares regression method as recommended by the IEEE Std-930 or the maximum likelihood method, which is often more convenient, since it is commonly used in commercial software. The probability of occurrence of each measurement is determined using a rank function f(i, n) and the one recommended in the IEEE standard is given by the following formula:

(11) $$f\left(i,n\right)=\frac{i-0.44}{n+0.25}*100\%$$

where *n* is the number of samples tested and *i* denotes the rank of the data in ascending order.

**Figure 10:**
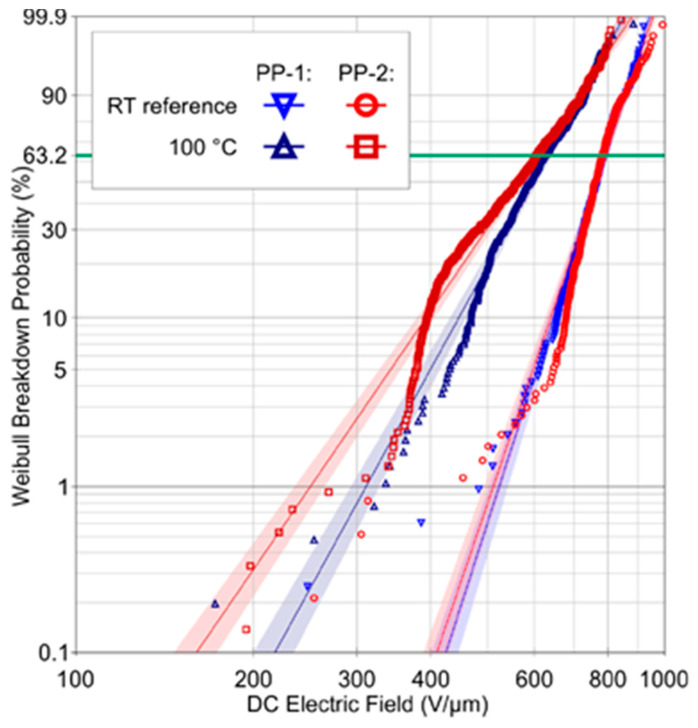
Weibull distribution of breakdown voltages at room temperature and at 100 °C for biaxially oriented PP (BOPP) film [26].

As a typical example of the dielectric strength measurement of a polymer film, Figure 10 shows the DC breakdown behavior at room temperature and at 100 °C of two industrially produced BOPP films, with different molecular weights labeled PP-1 and PP-2. The average thickness of the two films is 5.6 µm, with a standard deviation of 0.1 µm. The dielectric strengths at room temperature for both films with a 63.2% breakdown probability are 792 and 794 V/μm and 633 and 607 V/μm at 100 °C, respectively [26].

The technology currently used for manufacturing high-voltage and ultra-high-voltage capacitors uses coils placed in series, forming what is called the active part of the capacitor, which is impregnated in a synthetic oil, for example, during the manufacturing process. As previously explained in Section 2, the dielectric insulation system between two consecutive aluminum strips is composed of a mix of paper and dielectric film, with the paper having good compatibility with the impregnating oil. The electrical characteristics of film capacitors are directly linked to those of the dielectrics used.

Modern AC capacitors are usually wound using biaxially oriented polypropylene (BOPP) rolls in combination with thin aluminum foil. Depending on the application, other polymers, such as polyethylene (PE), polystyrene (PS), polypropylene (PP), polycarbonate (PC), Polyethylene naphthalate (PEN), Polyphenyl sulfide (PPS), polytetrafluorethylene (PTFE), polyester imides (PEI), polyethyleneterephthalate (PET), polybutyleneterephthalate (PBT), polyetheretherketone (PEEK), polyvinylchloride (PVC), polyvinylidene fluoride (PVDF), polyimides (PI), polyamides (PA) and polymethylmethacrylate (PMMA) can be used [7,9,11,27,28,29].

The three key dielectric properties needed when choosing a good film material for a capacitor are the dielectric constant or the relative permittivity, the dissipation factor and the breakdown strength [29].

The main drawback with using polymeric dielectric materials is their dielectric permittivity, which is relatively low (except for PVDF, others are generally <3), which explains their low energy densities. Using nanocomposites in the polymer manufacturing process improves the energy density in supercapacitor technologies [30].

For high-temperature applications, the thermal properties and how they are maintained at higher temperature are also critical. The polymer dielectrics currently used in capacitors for high temperature applications are polytetrafluoroethylene (PTFE), polycarbonate (PC), poly(ethylene terephthalate) (PET), poly(phenylene sulfide) (PPS), poly(ethylene 2,6-naphthalate) and polypropylene (PP):

- Polytetrafluoroethylene (PTFE) is thermally stable up to 260 °C and has a low dielectric constant close to 2.0 and a very low dissipation factor (DF) of less than 2 × 10^−4^ from 60 Hz to 3 GHz, since it is a non-polar material. It is used in applications where the maximum temperature is close to 200 °C and where low losses are required. Its crystalline melting temperature is 327 °C;
- Polycarbonate (PC) can be used in capacitors up to a temperature of 125 °C. Its dielectric constant is about 3 and it has a low DF of less than 0.001 at 1 kHz from 25 to 125 °C. Its maximum operating temperature is 125 °C and its glass transition temperature (Tg) is 150 °C;
- Poly(ethylene terephthalate) (PET) can be produced with a thickness as low as approximately 0.5 μm. At that thickness, its dielectric strength is around 500 MV/m. Its dielectric constant and operating temperature range are similar to those of polycarbonate, with its Tg being between 70 and 80 °C;
- Poly(phenylene sulfide) (PPS) is a semicrystalline polymer with a crystallinity of 60–65% and a crystalline melting temperature of 285 °C. It can be used up to 150 °C and its Tg is 85 °C;
- Poly(ethylene 2,6-naphthalate) (PEN) has the same ester functional group as PET and similar thermal and dielectric performance as PET. Its Tg is 125 °C;
- Polypropylene (PP) is the most widely used film for high-voltage and high-frequency applications. It can be fabricated with thicknesses down to 1.6 μm. It is a non-polar material and has a dielectric constant of about 2.2 and a very low DF of 10^−4^, which is essentially frequency-independent over several decades at temperatures below 100 °C. In the form of thin films, it has a dielectric strength of ~700 V/μm [31]. Its thermal conductivity in amorphous form is less than 0.2 W/(m K) and less than 0.3 W/(m K) in semicrystalline unoriented form. Its thermal conductivity in biaxially-oriented form is 0.6 W/(m K) and its maximum operating temperature is 105 °C [29];

Polymers such as fluorenyl polyester (FPE), polybenzobisthiazole (PBT), polybenzoxazole (PBO), polyquinoline (PQ), polybenzimidazole (PBI), poly(p-xylylene) (PPX), perfluoroalkoxy (PFA) and poly(4-methyl-1-pentene) (PMP) are also used in capacitor applications requiring even higher heat resistance [29];

- Fluorenyl polyester (FPE) has a glass transition temperature Tg of 335 °C and a dielectric constant of 3.5 at 25 °C with a 5% reduction at 300 °C. It has a dissipation factor (DF) of less than 0.003 between 100 Hz and 10 kHz and operates from 100 to 250 °C. Capacitors made with this type of polymer have shown a DF of 0.01 at 200 °C and a breakdown strength of 190 MV/m at 200 °C and 90 MV/m at 250 °C;
- PBO begins to decompose at around 600 °C. Its dielectric constant at 1 kHz is 2.8 and it operates from −55 to 300 °C. PBO has DF values of 10^−3^ at 50 °C and of 10^−2^ at 250 °C;
- Polyquinoline (PQ) has a Tg range of 300 to 400 °C. The dielectric constants of PBT and PQ at 1 kHz are 2.9 and 3.2, respectively. They operate from −55 to 300 °C. PBT and PQ exhibit a DF of about 0.01% at 25 °C at a frequency of 10 kHz. At high temperatures, the DF increases by 0.1% for the PBT and by 1% for the PQ at 250 °C;
- Polybenzimidazole (PBI) has a Tg of 435 °C. The dielectric constant and DF at 22 °C of its derivative polymer poly(2,2′-m-phenylene-5,5′-bibenzimidazole) (mPBI) are 3.4 and 0.02, respectively, from 60 to 100 kHz. The dielectric constant is close to 3.5 at 200 °C and 11 at 300° C, while the DF is close to 0.5 at 300 °C and 60 Hz;
- Poly(p-xylylene) (PPX) is a heat-resistant polymer. It is often used as a coating in printed circuit boards. It is obtained from the vapor phase polymerization of diradical monomers of para-xylylene (obtained by pyrolysis of 2,2-paracyclophane sublimated with a melting point (Tm) of 420 °C.). The dielectric constant and DF of parylene-N are close to 2.7 and 0.0015 at 22 °C, respectively, at frequencies ranging from 60 to 100 MHz. Its DF is 0.015 at 200 °C and 60 Hz;
- Perfluoroalkoxy (PFA) is a copolymer of tetrafluoroethylene and perfluoroalkyl vinyl ethers. It has a melting temperature of 305 °C and a dielectric constant of 2.1 at 22 °C and at a frequency of 60 Hz. Its DF values are 0.0001 at 60 Hz and 0.001 at 100 MHz at a temperature of 22 °C. Poly(4-methyl-1-pentene) (PMP) has a melting point of 233 °C. Its dielectric constant is close to 2.2 at 200 °C, with a DF of less than 0.0001 at 1 kHz and 10 kHz. Its use is limited due to its inability to fabricate thin film, having a thickness of 10 μm for capacitor applications [29].

Table 5 summarizes the thermal and dielectric properties of the most commonly used films in high-voltage capacitors.

For the manufacture of supercapacitors, the currently used polymers are polyaniline (PAn), polypyrole (PPY), polythiophene (PTh) and poly(p-phenylene vinylene) (PPV), among others. Given that the performance of supercapacitors depends on the interactions of the materials used in their manufacture (the combination of the material of the electrode and the type of electrolyte determines the thermal and electrical properties of these capacitors), these conductive polymers must have high electrical conductivity, low cost as compared to metal oxides, a fast charge–discharge rate and a high capacity and must be environmentally friendly. Their disadvantages are that they have poor mechanical stability and are highly conductive. Carbon is the most economical material for the manufacture of supercapacitors. Activated carbon (transformed by aqueous dispersions of polystyrene, ethylene, copolymers of acrylic acid) is the most used carbon in the manufacture of electrodes [21,22,23,24].

### 3.2. Nanocomposite Film

Polymer-based nanocomposites have several advantages due to their special properties, such as lightness, low cost and durability. They consist of two or more components, making up the matrix in which the nanometric particles (nanoparticles or nanofillers) constituting the second phase are dispersed. Nanocomposites can be divided into two groups: dielectric-dielectric nanocomposites, incorporating semiconductor nanofillers (TiO_2_, ZrO_2_, BaTiO_3_ (BT), CaCu_3_Ti_4_O_12_, Pb (Zr, Ti) O_3_, BN) with high dielectric permittivity, and dielectric nanocomposites conductors, incorporating conductive metal nanofillers (Ag, Cu) and conductive polymers (polyaniline, polypyrole). In the multilayer configuration, several high dielectric permittivity and high dielectric strength single-layer films are stacked in order to improve the energy density. One way to simultaneously increase the dielectric strength and the dielectric permittivity for nanocomposites is by fabricating multilayer dielectric films [30,46]. Graphene and carbon nanotubes (CNTs) are commonly used as fillers at the nanoscale as reinforcements in polymer matrix composites because they exhibit excellent mechanical properties. Using these reinforcements improves the electrical conductivity of polymer nanocomposites, and generally using these materials improves the thermal conductivity and allows high-temperature applications. The improvement of thermal conductivity depends on the nature and dispersion of the charges in the matrix [46].

### 3.3. Polymer Film Treatments

The surface treatment of films before their metallization and winding is used to promote their adhesion and improve their performance. The use of the corona treatment is standard for polypropylene. This technique allows different groups (hydroxyl, carbonyl and carboxylic groups) to be created on the surfaces of films, which increases the concentration of polar groups. The presence of these functional groups on a film’s surface in turn increases its free energy. In recent years, other treatments, such as plasma treatment with fluorine gases and vapors, ultraviolet irradiation and electron bombardment, have been carried out to modify the properties of film surfaces [3,6,11,47].

## 4. Encapsulant

The main role of the encapsulant is to consolidate and improve the dielectric strength of the capacitor assembly by filling any possible voids or air gaps, and thus improving the resistance to partial discharges [18]. In order to prevent faults inside capacitors using a liquid impregnant, the latter should always be in an airtight structure to prevent the loss of the impregnator. Most of the encapsulants used in high-voltage capacitors are polymers, which are initially in liquid form, oils or thermoset materials.

The oils used as an impregnation media for solid dielectrics have evolved over time, going from mineral oils to mono-di-benzyl-toluene (MDBT) and synthetic oils such as polychlorinated biphenyl (PCB), benzyl neocrapate (BNC) and mono-isopropyl-biphenyl (MIPB).

The most common liquid impregnants are mineral oils; vegetable oils such as castor, rapeseed and cottonseed oils; certain waxes; and biodegradable synthetic oils such as phenylxylylethane (PXE), monobenzyltoluene (MBT) and dibenzyltoluene (DBT) (Jarylec). These have completely replaced chlorinated biphenyls, which are currently prohibited due to environmental and health concerns.

Several mineral or synthetic oils are used to impregnate the dielectric. For example, trichlorodiphenyl (TCD) has been used as an impregnator on paper or mixed paper layer dielectrics. The PCB is replaced by synthetic aromatic hydrocarbon (AHC) oils, such as alkylnaphthalene (AN) and alkyldiphenylethane (ADE). Other non-flammable and low-permittivity oils such as silicone oils and alkylphenyl phosphate have also been used. Aromatic hydrocarbon (AHC) oils such AN and ADE both have excellent gas absorption properties, good compatibility and are environmentally safe. However, since AHC has low permittivity and is flammable, other insulating oils such as silicone phenylmethyl oil and mixtures of AHC and TCP are now partially used for power capacitors [14].

Liquid impregnants give excellent dielectric performance to capacitors, but ecological and safety requirements are directing research towards solutions using other impregnants, such as the use of thermosetting epoxy resins, plant-based oils and gases. If a solid impregnator is required, epoxy resins are available and represent a good alternative due to their excellent mechanical, electrical and chemical properties, as well as their advantageous processing possibilities in many applications [48]. Finally, dielectric gases can act as impregnators. The main gaseous impregnator used today is nitrogen (N_2_), which for certain applications is mixed with other gases such as sulfur hexafluoride (SF_6_) [9]. Other gases such as atmospheric air, CO_2_, fluoronitriles ((CF_3_)_2_CFCN) and fluoroketones CF_3_C(O)CF(CF_3_)_2_ (C5K ketone) can also be used. The dielectric strength of atmospheric air is 3 kV/mm and that of SF_6_ is three times higher than that of air, while the dielectric strength for fluoronitriles ((CF_3_)_2_CFCN) is twice that of SF_6_ and that of fluoroketones CF_3_C (O) CF (CF_3_)_2_ (C5K ketone) is 85% of the value of the dielectric strength of SF_6_ at −15 °C. The dielectric strength of the SF_6_ mixture (with a proportion of 20% SF_6_) and N_2_ is 70% of the value of the dielectric strength of SF_6_, while that of N_2_ is 44% of the value of the dielectric strength of SF_6_ [32,49,50,51,52].

To improve the properties of capacitors, composite polymers can be used. Polymer composites have several advantages, as they have the potential to improve the thermal conductivity and mechanical strength of materials. For the manufacture of polymer composites, polymeric materials such as epoxy resin are used as a matrix for advanced composites due to their ease of processing and low cost.

Nanodielectrics are a particular type of composite material, which as the name suggests, are dielectric materials that once dispersed in a polymer matrix influence the movement of polymer chains, leading to significant changes in the overall properties of the material [5]. Nanoparticles include SiO_2_, ZnO, Si_3_N_4_, BN and other oxides and nitrides, whose size range varies from a few nanometers to about 100 nm. To improve the properties of resins, low molecular weight organic and inorganic materials are often added [11].

It should be noted that the reasons why resins such as epoxies are used is mostly because they have high specific strength, efficient rigidity, chemical resistance and good dimensional stability. However, epoxy resin has certain drawbacks, namely poor thermal and electrical conductivity. To improve the unwanted properties of epoxies, the aforementioned nanocomposites are used to strengthen them and form epoxy composites. The materials used to this end are carbon nanotubes (CNT), silicon carbide nanowires, magnesium oxide, titanium dioxide and calcium carbonate. CNT are the most widely used due to their desirable properties, such as their high aspect ratios and good mechanical, electrical and thermal properties. Certain fillers, such as metallic particles, organic and inorganic particles, carbon, ceramics and fibers can also be added to polymers in order to improve their optical, thermal, electrical, mechanical and magnetic properties. The degree of improvement depends on how the charges disperse in the matrix and on the interfacial interactions between the different materials [46,53,54].

Table 6 and Table 7 give a list of the main encapsulating or impregnating materials used in manufacturing high-voltage capacitors, along with their dielectric properties.

## 5. Conclusions

In this literature review, we focused on the different technologies and materials used in manufacturing high-voltage capacitors. In the review, it is emphasized that different types of materials are used and their choices depend on the particular characteristics expected by the high-voltage capacitor manufacturer. For high-voltage applications, bi-oriented polypropylene (BOPP) is the most commonly used material. The most common liquid impregnants are minerals oils, and more recently epoxy, silicone and polyurethane resins.

The surface treatment of films, for example by corona treatment, before winding is done to promote their adhesion and improve their performance.

The use of certain gases, oils and resins in capacitors increases the emission of greenhouse gases into the atmosphere, as well as the risk of explosion due to the exposure of agents to oils during production. The desire to reduce carbon emissions has led to the production of capacitors and high-voltage equipment meeting environmental regulations. In order to overcome these problems, it is, therefore, important to meet the new challenges of dielectric materials. However, in order to participate in the preservation of the environment and to comply with environmental regulations, many industries today are investing in the use of more environmentally friendly materials that could be used in the high-voltage capacitors of tomorrow.

## Figures and Tables

**Table 2:** Comparison of the electrical and dielectric characteristics of power capacitors from 1950 to 1996.

Years	Manufacturing Technologies	Electric Field of Operation (V/μm)	Dissipation Factor (%)
1950–1959	All-paper capacitor using mineral oil	12	3.5
1960–1968	All-paper capacitor using PCB	16	2
1969–1974	Mixed capacitor using PCB	38	0.6
1975–1983	Mixed capacitor using non-chlorinated liquids	45	0.45
1984–1987	Mixed capacitor	45	0.45
1988–1996	All-film capacitor	60–75	0.1

**Table 3:** Main review articles focusing on metallized capacitor technologies.

**Technology**	Single- and double-stacked metallized capacitor	Wound metallized capacitor	Multisection metallized film
**References**	[12,13,14,15]	[2,14,15]	[2,16,17]

**Table 4:** Main review articles focusing on foil capacitor technologies.

**Technology**	Wound foil–film capacitor	Stacked foil–film capacitor	Hybrid film–foil metallized combination	Inserted tab capacitor	Multisection film–foil capacitor
**References**	[15]	[13]	[17]	[2,14]	[2,14,19]

**Table 5:** Summary of general characteristics of common dielectric polymer films [8,12,14,17,18,27,28,29,32,33,34,35,36,37,38,39,40,41,42,43].

Polymer Film	Dielectric Constant	Approximative Breakdown Strength (MV/m) ***	Dissipat-ion Factor at 1 kHz (%)	Operating Temperature (°C)	Meting Temperature (°C)
Polypropylene (PP)	2.2	640	0.02	−55 to 105	178
Polyester (PET)	3.3	570	0.5	−55 to 125	254
Polycarbonate (PC)	2.8	528	<0.15	−55 to 125	288–316
Polyphenylene sulfide (PPS)	3	550	0.05	−55 to 200	283
Polyvinylidene difluoride (PVDF)	12	590	<1.8	125	167–172
Polyethylene—naphthalate (PEN)	3	400	0.4	−55 to 137	266
Polyester (PEI)	3.3	550 at 22 °C	0.5	125	255
PTFE (Teflon)	2.1	296	0.05	260	327
Polystyrene	2.5	200	0.06	85	240
Polyethylene (PE)	2.3	500 (LDPE *)	<10^−2^	70 (LDPE *)	HDPE ** (135) LDPE * (110 to 115)
Polyvinyl Chloride (PVC)	4 5.3 (with a flexibilizer portion of 20 to 25%)	Operating field strengths are under 3 kV/mm	30 to 50 (with a flexibilizer portion of 20 to 25%)	60	100 to 260
Polyimides (PI)	2.88–3.48	22–27.6	0.01–0.03 (at 1Hz)	145–250 (maximum temperature)	250 to 300
Polyamide imides (PAI)	4.5 (PAI 1)	>85 (PAI 1)	<0.01 (PAI 1)	>200 (PAI 1)	-
Polysulfones (PSU) Polyethersulfones (PES)	low dielectric constant	high dielectric strength	Low dissipation factor	up to 150 and 200 high temperature and excellent low flammability	PSU: 400 PES: 340 up to 390
Polyamides (PA)	7 (PA 6) 4.5 (PA 12)	PA6: 24 to 30	300 (PA 6) 50 (PA 12)	75 (PA 6) 65 (PA 12)	220 (PA 6) 178 (PA 6)

Note: * low-density polyethylene; ** high-density polyethylene; *** the exact value of the dielectric strength is highly dependent on the measuring procedure (sample thickness, surrounding medium, rate of rise, type of electrode, etc.), and accordingly varies significantly from one reference to another [44,45].

**Table 6:** Overview of thermosetting and elastomer insulating materials [32,55,56,57].

Thermosetting	Relative Permittivity	tan δ	Conductivity (S/m)	Dielectric Strength (MV/m)
Epoxy Resins (EP)	3.5 to 4	<10^−2^	10^−12^ to 10^−15^	20
Polyurethanes (PU) (thermosetting)	4	2 × 10^−2^ (1 MHz)	10^−11^	-
Polyurethanes (PU) (elastomer)	7.4	>5 × 10^−2^ (1 MHz)	10^−10^ to 10^−12^	88.6
Phenolic Resin and Resin bonded Paper (RBP)	5	0.1 (1 MHz)	10^−11^	-
Silicone	2.8 to 3	0.005 to 0.01	10^−13^	26–36

**Table 7:** Overview of insulating liquids [32,58,59,60,61,62,63,64].

Liquids	Relative Permittivity	tan δ	Dielectric Breakdown, (kV)	Fire Point (°C)	Flash Point (°C)
Mineral oil	2.2 at 20 °C	<0.05 (25 °C)	30–85	170	145
Silicone oils	2.7 at 20 °C 2.3 at 200 °C	1 to 2.10^−4^	35–60	>335	>300
Ester liquids	3.3	≥10^−3^	45–70	257	310
Vegetable oils	3.1	0.25 (25 °C)	82–97	354–360	310–325
Natural Ester Liquids	3.1 at 20 °C	≤0.2 at 25 °C	33.8	300	275
Natural Ester Liquids	3.1 at 20 °C	≤0.2 at 25 °C	33.8	300	275

## Data Availability

The data presented in this study are available on request from the corresponding author.
